# Radiomics in Urolithiasis: Systematic Review of Current Applications, Limitations, and Future Directions

**DOI:** 10.3390/jcm11175151

**Published:** 2022-08-31

**Authors:** Ee Jean Lim, Daniele Castellani, Wei Zheng So, Khi Yung Fong, Jing Qiu Li, Ho Yee Tiong, Nariman Gadzhiev, Chin Tiong Heng, Jeremy Yuen-Chun Teoh, Nithesh Naik, Khurshid Ghani, Kemal Sarica, Jean De La Rosette, Bhaskar Somani, Vineet Gauhar

**Affiliations:** 1 Department of Urology, Singapore General Hospital, Singapore 169608, Singapore; 2 Urology Unit, Azienda Ospedaliero-Universitaria Ospedali Riuniti di Ancona, Università Politecnica Delle Marche, 60126 Ancona, Italy; 3 Yong Loo Lin School of Medicine, National University of Singapore, Singapore 119077, Singapore; 4 Department of Urology, National University Hospital, Singapore 119074, Singapore; 5 Department of Urology, Saint-Petersburg State University Hospital, 199034 St. Petersburg, Russia; 6 Department of Urology, Ng Teng Fong Hospital, Singapore 609606, Singapore; 7 S.H. Ho Urology Centre, Department of Surgery, The Chinese University of Hong Kong, Hong Kong SAR, China; 8 Department of Mechanical and Industrial Engineering, Manipal Institute of Technology, Manipal Academy of Higher Education, Manipal 576104, Karnataka, India; 9 Department of Urology, University of Michigan, Ann Arbor, MI 48109, USA; 10 Department of Urology, Biruni University, 34010 Istanbul, Turkey; 11 Istanbul Medipol University, TEM Avrupa Otoyolu Goztepe Cikisi No: 1, 34010 Istanbul, Turkey; 12 Department of Urology, University Hospital Southampton NHS Foundation Trust, Southampton SO16 6YD, UK

**Keywords:** radiomics, urolithiasis, therapeutic applications

## Abstract

Radiomics is increasingly applied to the diagnosis, management, and outcome prediction of various urological conditions. Urolithiasis is a common benign condition with a high incidence and recurrence rate. The purpose of this scoping review is to evaluate the current evidence of the application of radiomics in urolithiasis, especially its utility in diagnostics and therapeutics. An electronic literature search on radiomics in the setting of urolithiasis was conducted on PubMed, EMBASE, and Scopus from inception to 21 March 2022. A total of 7 studies were included. Radiomics has been successfully applied in the field of urolithiasis to differentiate phleboliths from calculi and classify stone types and composition pre-operatively. More importantly, it has also been utilized to predict outcomes and complications after endourological procedures. Although radiomics in urolithiasis is still in its infancy, it has the potential for large-scale implementation. Its greatest potential lies in the correlation with conventional established diagnostic and therapeutic factors.

## 1. Introduction

The exponential growth of medical digitalization and data acquisition has led to the healthcare sector embracing artificial intelligence (AI) to manage and optimize data accruement and utilization [1]. The scope of analysis has correspondingly broadened and introduced a new scientific field collectively called “omics” [2]. The branches of science known informally as omics refers to a field of study in biological sciences that ends with -omics, such as genomics, transcriptomics, proteomics, or metabolomics. The application of AI capabilities within the context of medical imaging is known as radiomics. Radiomics is a quantitative method that primarily extracts extensive amounts of mineable data from medical imaging and radiographic images [3]. These features are subsequently input into statistical frameworks and evaluated. It quantifies textural information by using analysis methods from the field of artificial intelligence to analyse “big data” [4]. Big data is defined as “a term that describes large volumes of high velocity, complex and variable data that require advanced techniques and technologies to enable the capture, storage, distribution, management, and analysis of the information” [5]. AI is used for mathematical extraction of the spatial distribution of signal intensities and pixel interrelationships and quantifies textural information which is otherwise imperceptible to humans [6,7]. Radiomics aims at improving precision medicine by using AI to improve diagnostic and prognostic information [8,9]. It surpasses the human ability to identify key imaging characteristics imperceptible to the naked human eye, picking up hidden objective data that may influence subsequent treatment decisions [10].

Data-characterization algorithms such as machine learning (ML), deep learning (DL), and artificial neural networks (ANNs) have already been incorporated to generate radiomics-guided learning models that guide diagnosis, stratification, and treatment [11,12]. In recent years, radiomics has been increasingly applied to the diagnosis, management, and outcome prediction of several medical and urological conditions.

**Figure 1:**
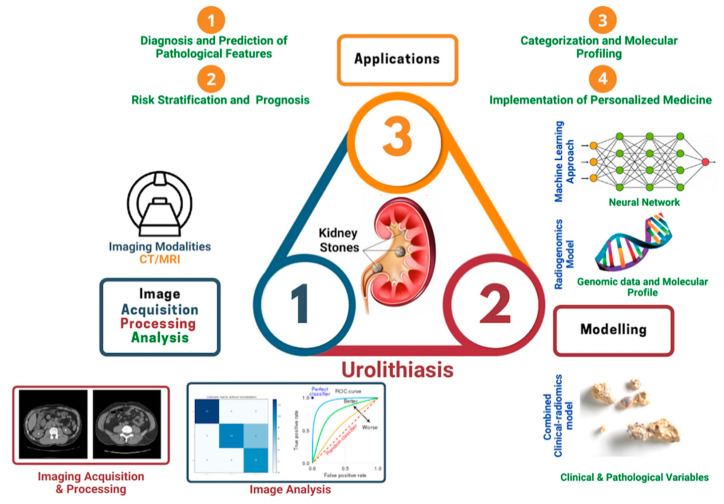
Potential contributions of radiomics and radiogenomics to the management of a patient with urolithiasis.

**Figure 2:**
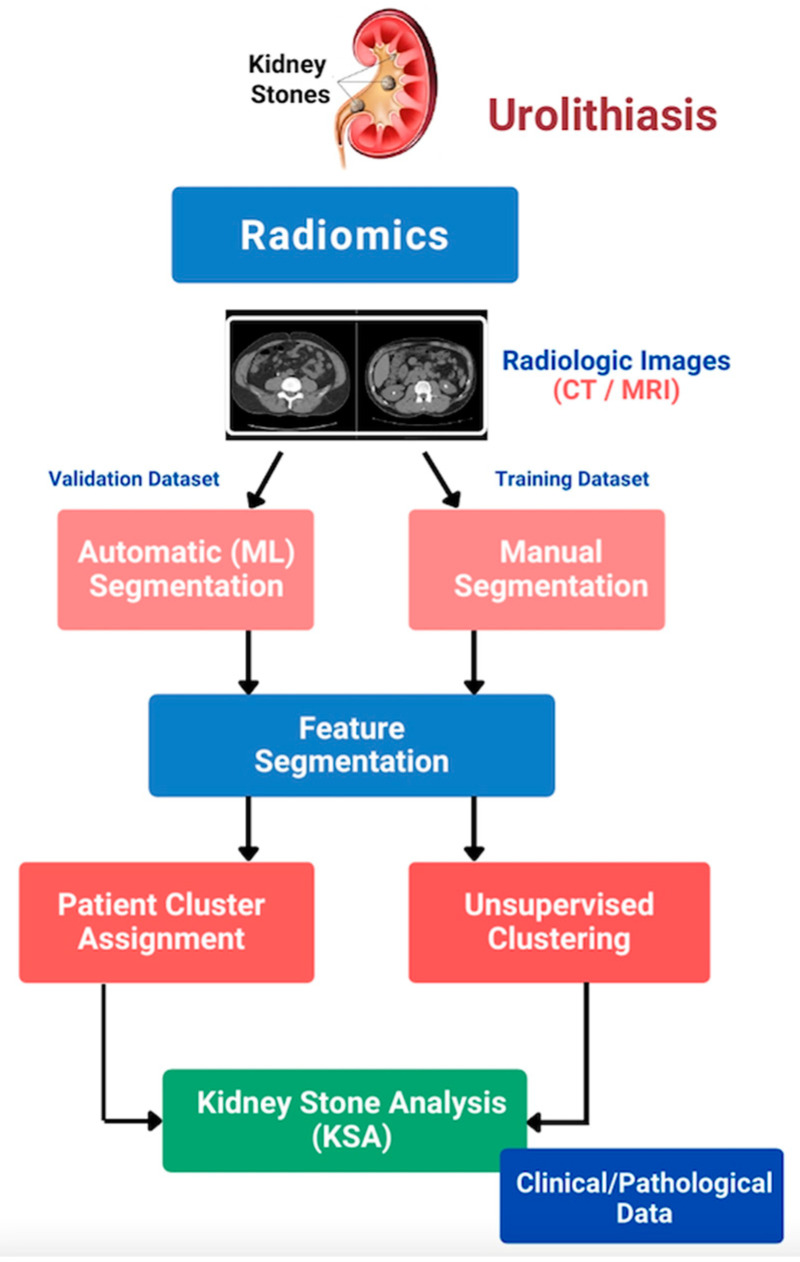
Radiomics approach in the treatment of patients with kidney stone disease.

First utilized in oncology, radiomics has been successfully investigated to differentiate benign renal mass from malignancy and predict histopathology, survival, and outcome of various urologic cancers [13]. Radiomics aims to analyse and translate medical images into quantitative data and provide an image-based biomarker to aid clinical decisions and improve precision medicine [14]. Success in the oncologic field has drawn attention to the application of radiomics in benign urologic conditions, especially urolithiasis. An illustration of the workflow of radiomics in kidney stone disease is presented in Figure 1: (1) Image acquisition and pre-processing, (2) Validation and training dataset creation, (3) Extraction and feature segmentation, and (4) Model building, e.g., kidney stone analysis. Figure 2 illustrates the utility of radiomics when applied specifically to patients with kidney stone disease, with four potential key areas: (1) Diagnosis and prediction of pathological features in patients with kidney stone disease, (2) Risk stratification and prognosis of stone forming patients, (3) Categorisation and molecular profiling of high-risk stone formers; and (4) Implementation of personalized medicine in kidney stone formers.

The aim of the scoping review is to evaluate if radiomics-based applications can help endourologist overcome some confounders in stone management such as preoperative identification of stone composition, identifying phleboliths, and predicting stone free rate after medical expulsion therapy.

## 2. Materials and Methods

### 2.1. Literature Search

A literature review of the usage of radiomics in the setting of urolithiasis was performed using the Preferred Reporting Items for Systematic Reviews and Meta-Analyses (PRISMA) framework for scoping reviews and metanalysis guidelines. An electronic literature search was conducted on PubMed, EMBASE, and Scopus from inception to 21 March 2022 without language restrictions (Appendix A) [15]. The full search strategies are outlined in Appendix B. Abstracts and full texts retrieved were reviewed by two independent investigators; conflicts were resolved by a third author. The inclusion criteria were: (1) Use of radiomics in diagnostics, treatment prediction, or therapeutics; (2) Any type of urolithiasis, including nephrolithiasis, ureterolithiasis, and cystolithiasis. Case reports, abstracts, and reviews were excluded from the analysis. The data were extracted using a standardized data collection template with predefined data fields including study characteristics, objective of radiomics, study findings, and study conclusions.

### 2.2. Study Selection

The search strategy retrieved 1332 studies; after removal of 322 duplicates, the remaining 1010 studies were screened by title and abstract. Of the 108 studies shortlisted for full-text screening, 7 were eventually included in this review.

## 3. Results

The potential domains in which radiomics can contribute significantly are diagnostics, therapeutic, and interventional outcomes. A total of seven studies were included; one study by Perrot et al. [16] examined the use of radiomics in differentiating between phleboliths and calculi. Four studies reviewed identified stone type and composition, with two studies that looked at interventional outcomes [17,18]. Table 1 shows the included studies. 

## 4. Discussion

### 4.1. Diagnostics

#### 4.1.1. Differentiating Ureteric Calculi and Phleboliths

First described in the 19th century, phleboliths generally present as layers of calcified fibrous tissue covered by a layer of endothelium which is continuous with the intimal layer of vein wall [19]. Differentiating characteristics include a central lucency, comet tail sign, and anatomical distribution [20]. Although advances in radiology have improved the landscape of differentiating phleboliths from ureteral calculi, they still present a diagnostic challenge particularly in the emergency setting, leading to unnecessary intervention and associated financial and resource burdens. Perrot et al. [16] sought to utilize the capabilities of radiomics to improve the use of low-dose unenhanced computed tomography (LDCT) in distinguishing renal calculi from pelvic phleboliths. The study involved independent training (369 patients, 211 kidney stones, and 201 phleboliths) and a testing cohort (43 patients, 24 kidney stones, and 23 phleboliths) for training and experimentation of the machine-learning classifier, respectively. Both patient groups presented with acute renal colic and subsequently underwent LDCT for radiological assessment. A total of 147,029 radiomics features (first-order, shape, gray level co-occurrence matrix (GLCM), gray level size zone matrix (GLSZM), gray level run length matrix (GLRLM), neighboring gray-tone difference matrix (NGTDM), and gray level dependence matrix (GLDM)) extracted from LDCT images were used for prediction by the model, demonstrating an overall accuracy of 85.1%, 91.7% sensitivity, and 78.3% specificity with a ROC-AUC value of 0.902. This radiomics-reinforced machine-learning algorithm proves itself to be a highly objective method for discerning renal calculi and might be helpful in limiting unnecessary interventions. 

#### 4.1.2. Pre-Operative Identification of Stone Type

Radiomics features have also been employed to guide the detection of calculi material, largely within the pre-operative context to guide downstream management. Cui et al. [21] developed a radiomics signature created with ensemble learning based on bagged trees and applied it to non-contrast CT images of 157 patients diagnosed with either infection stone (98 patients) or non-infection stone (59 patients). With the least absolute shrinkage and selection operator (LASSO) algorithm, 27 radiomics features with the highest predictabilities were selected. The model reported 90.7% accuracy, 85.8% sensitivity, and 94.0% specificity with a ROC value of 0.97 in determining the presence of infection kidney stones. In the same vein, Zheng et al. [22] established a radiomics-signature incorporated radiomics model after extraction of data from CT images of 1198 urolithiasis patients, with 24 best radiomics features finalized by LASSO from 1316 radiomics features. AUC values of 0.898 (95% CI 0.840–0.956), 0.832 (95% CI 0.742–0.923), 0.825 (95% CI 0.783–0.866), and 0.812 (95% CI 0.710–0.914) were attained with the model on training and validation cohorts. The model also performed significantly better (*p* < 0.001) than urine pH, urine white blood cell count, urine nitrite, and presence of urease-producing bacteria in determining the existence of infection renal stones. 

Tang et al. [23] specifically looked at the prediction of the occurrence of calcium oxalate monohydrate (COM) stones, the most prevalent stone type in routine practice. A total of 1218 radiomics features were extracted from 337 COM and 107 non-COM calculi seen on pre-operative non-contrast CT images, and 8 with non-zero coefficients were selected for the model by LASSO. Incorporation into the AI model revealed an accuracy, sensitivity, and specificity of 88.5%, 90.5%, and 84.3%, respectively, with an AUC value of 0.935 (95% CI 0.907–0.962) in the training cohort and 0.933 (95% CI 0.893–0.973) in the testing set for pre-operative prediction of COM vs. non-COM stones. Hameed et al. [24] applied deep learning convolutional neural network (DLCNN) guided by radiomics features demonstrating 87% accuracy of prediction of calculi type. Specificity of each type of calculi was 89% for COM stones, 85% for calcium oxalate dihydrate stones, 86% for struvite stones, 93% for uric acid stones, and 89% for calcium hydrogen phosphate stones. However, despite improvements with added anatomical location and the ability to aid in differentiating between pelvic phleboliths and ureteric calculi, there are still sizable inaccuracies if artificial intelligence is used alone. Like AI, radiomics too can efficiently process vast quantities of data. With the shift towards electronic patient records, increasingly more big data sets are created and this will allow AI and radiomics to analyse and detect novel diagnostic and treatment patterns in the future [25].

Summary: With increasing application and accuracy of radiomics in differentiating phleboliths from true calculi and stone type, this can potentially influence the choice of treatment modality and limit unnecessary surgical intervention with its associated financial burden and morbidity.

### 4.2. Evaluating Treatment Outcomes

Radiomics has been also applied in the field of urolithiasis to predict the complications and outcomes of endourological procedures. We review their role in the various treatment modalities in predicting treatment efficacy.

#### 4.2.1. Prediction of Spontaneous Stone Passage

Radiomics has also been applied to predict spontaneous stone passage rate in symptomatic patients. Mohammadinejad et al. compared the ability of a semi-automated radiomics analysis software in predicting the likelihood of spontaneous stone passage with manual measurements. Stone characteristics including length, width, height, maximal diameter, volume, the mean and standard deviation of the Hounsfield units, and morphologic features were extracted from CT images using automated radiomics analysis software [26]. Univariate analysis and multivariate analysis showed AUC of 0.82 and 0.83, respectively, for maximum stone diameter measured manually. The AUC for a model including automatic measurement of maximum height and diameter of the stone was 0.82. Hence, the authors concluded that semi-automated radiomics analysis shows similar accuracy compared with manual measurements in predicting spontaneous stone passage.

#### 4.2.2. Therapeutic Utility in ESWL

Despite numerous AI-based platforms exploring the utility of decision algorithms in ESWL, there were no articles that focused specifically on radiomics devised applications for ESWL, proving it to be uncharted therapy. It will be interesting to continue to monitor if refined technologies such as burst wave lithotripsy will fuel renewed interest in the application of radiomics in this modality of treatment [27].

#### 4.2.3. Predicting Stone Burden Affecting RIRS/PCNL Stone-Free Rates Outcomes

The application of radiomics in endourology is relatively novel, and only two reports have been so far published, one in PCNL [17] and the second one in RIRS [18]. Homayounieh et al. analysed 202 kidney stone adult patients who underwent CT scan for evaluation of renal colic or stones in three different CT machines [2]. The purpose of this study was to assess if an automatically segmented whole renal radiomics was able to estimate the stone burden and predict hydronephrosis and treatment strategies from CT images. All stone images were evaluated by a single experienced radiologist who assessed manually the stone location and burden (stone density, stone size, and stone contours) and the presence or not of hydronephrosis for each patient. A physician expert in image processing processed all CT examinations from a standalone radiomics prototype that automatically recognized and segmented the entire kidney volume, including all stones included within the segmentation contours. After confirmation of the contours, the radiomics prototype estimated 1690 first-, shape, and higher-order radiomics for each kidney. Among the 202 patients, the radiomics prototype was able to discriminate between patients with and without renal stones (AUC 0.84, 95% CI 0.78–0.89, *p* < 0.003). Radiomics was also able to accurately detect hydronephrosis (AUC 0.89, 95% CI 0.8–0.89, *p* < 0.003). In addition, radiomics was able to predict patients managed with PCNL. Stone burden in these patients was significantly larger than those managed conservatively (641 ± 1090 vs. 53 ± 8 mm^3^, *p* < 0.0001). Interestingly, there was no difference in radiomics vendors performance between the three CT machines across all study outcomes. The automatic segmentation and inclusion of the entire kidney volume enabled the authors to apply radiomics not only to the stone but to the whole renal volume to obtain a consistent and generalizable prediction of stone burden and the need for PCNL treatment.

Factors such as location [28], size and volume of stone burden [29,30], and Hounsfield units (HU) [31] are key determinants and predictors of stone-free rates in both normal and anomalous kidneys alike [32,33]. Stone size limits the use of HU for the prediction of stone composition, especially calcium oxalate stones, and is a known limitation for predicting successful outcomes in ESWL and PCNL [34,35] Xun et al. retrospectively assessed 264 patients with a solitary kidney stone who underwent RIRS [18]. Among these, 142 patients had a lower calix stone. Preoperative assessment was made with an unenhanced 64-slice CT scan. Stones were manually segmented on each transverse slice CT image. Radiomics feature extraction was accomplished operating an in-house texture analysis software, including a total of 604 radiomics features (first-order statistics, shape- and size-based features, textural features, and wavelet features) generated from each original CT image. A radiomics signature was generated by a linear combination of selected features weighted by their respective coefficients, and a radiomics score (Rad-score) was estimated for each patient. Finally, the authors developed a visual nomogram incorporating clinical and radiomics parameters to predict SFR, defined as residual fragments less than 2 mm. Interestingly, radiomics score significantly differed between SFR and non-SFR patients both in the test and validation group with higher scores observed in patients with higher SFR. The prediction nomogram was very accurate (AUC 0.94, 95% CI, 0.910–0.989) and its predictive efficacy was confirmed by the validation group (AUC 0.947, 95% CI 0.883–1). The inclusion of radiomics in this model demonstrated to be an effective pre-operative prediction method for clinical decision-making in patients undergoing RIRS. The main advantage of using radiomics in this context relies mostly on the speed of the procedure in a more quantitative and reproducible manner as compared with the manual assessment which can be time-consuming and prone to intra and interobserver variations, particularly for complex renal stones requiring a PCNL treatment (i.e., staghorn and multiple stones) [36].

Summary: This adds a significant research potential wherein using the radiomics signatures comparisons can be made between the efficacy of single emission CT scans (SECT) vis a vis dual emission CT scans to accurately determine stone composition [37]. This information can enable endourologists to better choose the right intervention for their patient and potentially overcome limitations and act as adjuncts of various scoring systems used as surrogate tools for predicting success in endourology interventions [38].

### 4.3. Current Limitations and Future Directions

One limitation of deep learning-based radiomics is the dependent correlation between the features and the input data, as the features are generated from that very dataset. Therefore, in contrast to feature-based radiomics, large datasets are necessary to accurately identify the relevant and robust feature subsets. This limitation can be partially overcome by utilizing a machine learning technique called transfer learning, by using a pre-trained neural network on a different but similarly related task, e.g., Neural data that was trained to predict renal stones can also be used and trained on how to measure and classify residual fragments after a procedure [39]. Another limitation is the reproducibility and transferability of radiomics features as it is heavily dependent on size, quality, sequence, modality, resolution, and motion artifacts of image transfer; Traverso et al. performed a recent review and identified radiomics features that were reproducible and repeatable [40]. Moving forward, the Image Biomarker Standardization Initiative (IBSI) has been established to provide standardized image biomarker nomenclature and definition, as well as to aid in formulating reporting guidelines to regulate effective communication and verification within study groups in the field of radiomics [41]. These principles when applied to the field of radiomics for urolithiasis could help standardize and refine accessibility, facilitating a widespread acceptance of the same. Xun et al. developed and validated a clinical-radiomics nomogram model for pre-operatively predicting the stone free rate of flexible ureteroscopy. They demonstrated that when applied, radiomics scores from their nomogram had satisfactory predictive accuracy in clinical application [18]. Radiomics may be used in the future to generate or validate nomograms that aid in accessing or predicting stone-free rates based on the modality of intervention.

### 4.4. Take Home Messages

In summary, potential applications of radiomics in urolithiasis are:

1. Predicting success of spontaneous stone passage with medical expulsion therapy.
2. Differentiating between calculi and phleboliths.
3. Pre-operative accurate identification of stone type.
4. Predicting stone burden affecting RIRS/PCNL stone-free rate outcomes.

## 5. Conclusions

Our review shows that radiomics in urolithiasis is still in infancy. Its best potential lies in identifying infectious stones preoperatively; whether this application can extend to all stone types remains undetermined. Future applications in ESWL and predicting stone free rates for different compositions are the next frontiers for research and development. It is hoped that with further correlation of radiomics with conventional established sources of diagnostic subsets such as clinical, molecular, and imaging can optimize disease management in urolithiasis and improve patient prognosis. 

## Figures and Tables

**Table 1:** Summary of included studies.

No.	Author (Year)	Type of Study	Objective	Number of Patients and Breakdown	Number of Radiomics Features	Utility	Conclusion
1	Perrot et al., (2022)	In-vivo	Identification of Urolithiasis	Training set: 369 patients (211 kidney stones, 201 phleboliths) Testing set: 43 patients (24 kidney stones, 23 phleboliths)	NR	Accuracy: 85.1% Sensitivity: 91.7% Specificity: 78.3% Positive predictive value: 81.5% Negative predictive value: 90.0% AUC: 0.902	Machine learning reinforced with machine learning enable accurate discernment between renal calculi and phleboliths on low-dose CT in patients with acute flank pain.
2	Cui et al., (2022)	In-vivo	Prediction of Stone Type	157 patients (98 infection kidney stones, 59 non-infection kidney stones)	54 radiomics features (16 morphological, 38 textural) → reduced to 27 key features (16 morphological, 11 textural) by the LASSO algorithm	Accuracy: 90.7% Sensitivity: 85.81% Specificity: 93.96% Positive predictive value: 91% Negative predictive value: 91% AUC: 0.97	Quantitative nomogram with radiomics method is useful for pre-operative prediction of infection versus non-infection kidney stones.
3	Zheng et al., (2022)	In-vivo	Prediction of Stone Type	Training set: 314 patients (41 infection stones, 273 non-infection stones) Internal validation set: 134 patients (22 infection stones, 112 non-infection stones) External validation set 1: 594 patients (111 infection stones, 483 non-infection stones) External validation set 2: 156 patients (18 infection stones, 138 non-infection stones)	1316 radiomics features → 24 key features with non-zero coefficients selected by the LASSO algorithm	Training set: AUC: 0.864 (95% CI 0.802–0.926) Internal validation set: 0.832 (95% CI 0.742–0.923) External validation set 1: 0.825 (95% CI 0.783–0.866) External validation set 2: 0.812 (95% CI 0.710–0.914)	Radiomics model developed can be a non-invasive method to detect urinary infection stones in vivo, benefitting subsequent management and patient prognosis.
4	Tang et al., (2022)	In-vivo	Prediction of Stone Composition	543 patients (373 calcium oxalate monohydrate stones, 170 non-COM stones)	1218 radiomics features extracted → 8 features with non-zero coefficients were selected for by the LASSO algorithm	Accuracy: 88.5% Sensitivity: 90.5% Specificity: 84.3% Training set AUC: 0.935 (95% CI 0.907–0.962) Testing set AUC: 0.933 (95% CI 0.893–0.973)	Artificial intelligence models incorporated with radiomics can predict COM and non-COM stones in vivo pre-operatively with robust accuracy, sensitivity, and specificity values.
5	Hameed et al., (2022)	In-vitro	Prediction of Stone Composition	NR	NR	Average accuracy: 87% Calcium oxalate monohydrate stone accuracy: 89% Calcium oxalate dihydrate stone accuracy: 85% Struvite stone accuracy: 86% Uric acid stone accuracy: 93% Calcium hydrogen phosphate stone accuracy: 89%	The artificial intelligence (deep learning-convolutional neural network DL-CNN) model reinforced with radiomics is successful in predicting various types of stone composition with high accuracy values.
6.	Xun et al., (2020)	In-vivo	PCNL: To develop and validate a novel clinical–radiomics nomogram model for pre-operatively predicting the stone-free rate of flexible ureteroscopy in patients with a single kidney stone	Training set: 99 patients Testing set (internal validation): 43 patients	Radiomics feature selection and signature building were conducted by using the least absolute shrinkage and selection operator (LASSO) method. With penalty parameter tuning conducted by 10-fold cross-validation, LASSO was performed to select robust and non-redundant features from the primary cohort. A radiomics signature was created by a linear combination of selected features weighted by their respective coefficients, and the relevant radiomics score was calculated for each patient.	AUC test group: 0.949 (95% CI, 0.910–0.989) AUC validation group: 0.947 (95% CI, 0.883–1)	Radiomics score, stone volume, hydronephrosis level, and operator experience were crucial for RIRS strategy
7.	Homayounieh et al., (2020)	In-vivo	RIRS: To assess if auto segmentation-assisted radiomics can predict disease burden, hydronephrosis, and treatment strategies in patients with renal calculi.	202 patients who underwent clinically indicated, non-contrast abdomen-pelvis CT for suspected or known renal calculi.	Deidentified CT images were processed with the radi- omics prototype (Radiomics, Frontier, Siemens Healthineers), which automatically segmented each kidney to obtain 1690 first-, shape-, and higher-order radiomics.	AUC: 0.91 (95% CI 0.85–0.92)	Automated segmentation and radiomics of entire kidneys can assess hydronephrosis presence, stone burden, and treatment strategies for renal calculi

NR: Not reported; AUC: Area under the curve; CT: Computed Tomography; PCNL: Percutaneous nephrolithotomy; RIRS: Retrograde intrarenal surgery.

**Figure A1:**
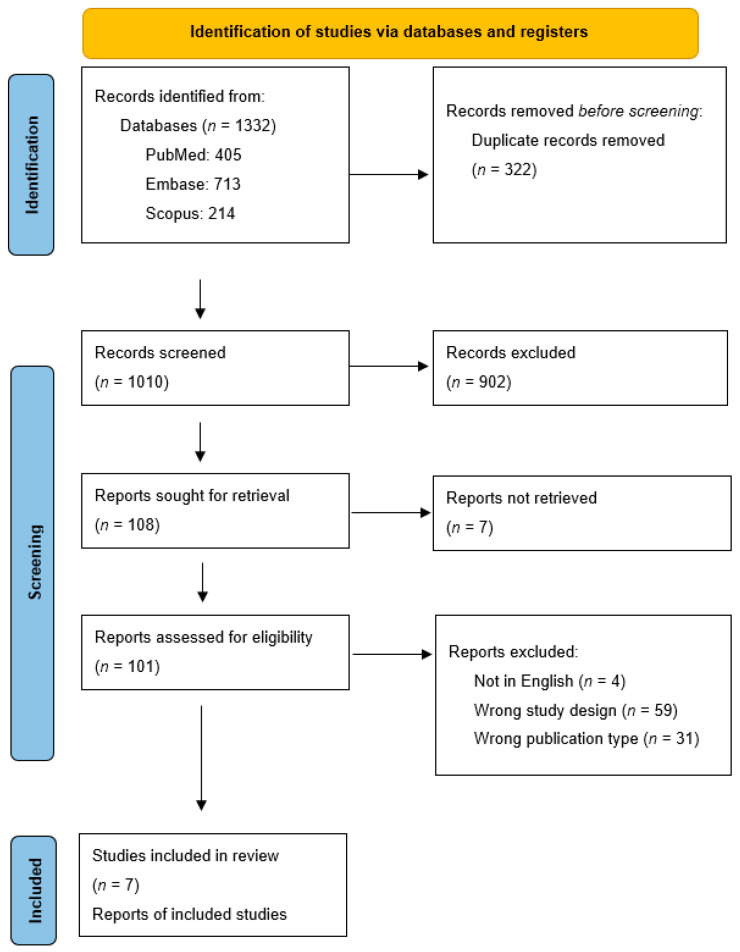
PRISMA 2020 Flow Diagram.

## Data Availability

Not applicable.

## Appendix B

**Table A1:** Full search phrases used for the respective databases.

PubMed	405 Articles
((stone * AND (renal OR kidney OR ureter OR ureteric OR bladder)) OR (‘Urolithiasis’ [MeSH]) OR (‘Calculi’ [MeSH]) OR (‘Kidney Calculi’ [MeSH]) OR nephrolithiasis OR ureterolithiasis OR cystolithiasis) AND (“artificial intelligence” [MeSH] OR “AI” OR “radiomic *” OR “machine learning” OR “deep learning”)	
EMBASE	713 articles
((stone OR stones) AND (renal OR kidney OR ureter OR ureteric OR bladder) OR ‘urolithiasis’/exp OR ‘calculi’/exp OR ‘nephrolithiasis’/exp OR ‘ureterolithiasis’/exp OR cystolithiasis) AND (‘artificial intelligence’/exp OR ‘ai’ OR ‘radiomic’ OR ‘radiomics’/exp OR ‘machine learning’/exp OR ‘deep learning’)	
Scopus	214 articles
TITLE-ABS-KEY (((stone OR stones OR calculi OR calculus AND (renal OR kidney OR ureter OR ureteric OR bladder)) OR urolithiasis OR nephrolithiasis OR ureterolithiasis OR cystolithiasis) AND (“artificial intelligence” OR “AI” OR “radiomic” OR “radiomics” OR “machine learning” OR “deep learning”))	

Date searched: 21 March 2022. Pre-deduplication 1332. Post-deduplication 1010.
